# An unusual case of Adrenocortical Adenocarcinoma with Biochemical Masquerade of Pheochromocytoma

**DOI:** 10.12669/pjms.37.4.3916

**Published:** 2021

**Authors:** Muzaffar Ali, Lubna Mirza

**Affiliations:** 1 Muzaffar Ali, Rehman Medical Institute, Peshawar, Pakistan; 2 Lubna Mirza Norman Regional Hospital, Norman, United State

**Keywords:** Adenocarcinoma, Adrenocortical, Tumor, Metastases, Adrenalectomy Pheochromocytoma

## Abstract

Adrenocortical carcinoma (ACC) is a rare malignancy that arises from the adrenal cortex and often presents as adrenal incidentaloma on abdominal scans with rise in the use of imaging modalities. ACC often presents as Cushing’s syndrome or virilization. On the other hand, pheochromocytoma is an adrenal medullary tumor. It is rare for ACC to present as pheochromocytoma even though both may coexist. Moreover, ACC tumors have radiological and histological features suggestive of aggressive nature of the disease.

We present a case of a 65-year-old lady who initially presented with a 3cm left adrenal incidentaloma. All of her adrenal hormones were in normal range. She was lost to follow up for several years and returned with a much enlarged lesion. Biochemical work up showed mildly increased catecholamines and metanephrines suggestive of pheochromocytoma. She didn’t have any signs or symptoms of pheochromocytoma. She was treated with alpha blockers before surgery as a prophylactic measure. Surgical pathology was consistent with the diagnosis of primary adrenal adenocarcinoma. We recommend that adrenal incidentalomas should be followed annually for up to five years as per American association of Endocrinology and the Endocrine Society guidelines to prevent morbidity and mortality in patients.

## INTRODUCTION

Adrenocortical carcinoma (ACC) is an extremely rare entity with a reported incidence of 0.5-2 cases per million/year and can account for up to 14% cases referred as incidentaloma. Approximately 60 percent of ACCs are sufficiently secretory to present clinical syndrome of hormone excess.

ACC is one of the major indications for adrenalectomy in cases of adrenal incidentaloma. ACC can present in a variety of ways with clinical features consistent with Cushing’s and virilization being the most common^1^, with overproduction of both glucocorticoids and androgens.

The clinical manifestations related to tumor growth (i.e., abdominal or flank pain) maybe the main presenting complaint in most patients with nonfunctioning tumors (or more precisely with subclinical production of steroids), or with an incidentally found adrenal mass detected on radiographic imaging. The evaluation in asymptomatic patients has been debated.

Computed tomography (CT) scanning can usually distinguish adenomas from ACCs. Magnetic resonance imaging (MRI) is complementary to CT scan, in that local invasion and involvement of the surrounding major vasculature are more readily identifiable. The maximum diameter of the adrenal mass is predictive of malignancy. Most adrenal adenomas are less than 4-cm in diameter. In contrast, most ACCs are greater than 4cm in diameter when discovered. Cytology from a specimen obtained by fine-needle aspiration (FNA) cannot distinguish a benign adrenal mass from adrenal carcinoma. It can, however, distinguish between an adrenal tumor and a metastatic tumor.

Pheochromocytoma is another rare neoplasm occurring in 0.1% of hypertensive patients.^1^ It is exceedingly rare for ACC and pheochromocytoma to exist concurrently. This concurrence has been reported only a few times in the literature. Pheochromocytoma should always be excluded by biochemical testing before attempting biopsy.

## CASE REPORT

A 62-year-old Caucasian woman presented to our endocrinology clinic for the evaluation and treatment for Osteoporosis. She had a history of a recent fragility hip fracture requiring surgery.

Bone density scan/ Dual-energy X-ray absorptiometry (DEXA scan) showed worst score of -2.7 in the left femoral neck which was consistent with the diagnosis of Osteoporosis. During her interview, she mentioned a 3cm left sided adrenal tumor that was discovered incidentally by her Hematology/Oncology team. She had undergone bilateral salpingo-oophorectomy and hysterectomy for a left pelvic mass measuring 5 cm x 4 cm which was suspicious for neoplasm but later found to be benign on post-surgical pathology.

One of her abdominal CT scans done during her oncology follow-ups showed a 3.0 x 2.4 cm left adrenal mass. The Hounsfield Units (HU) values of the mass were 37 HU pre-contrast, 125 HU post-contrast, and 74 HU during the delayed contrast phase. The enhancement characteristics of the lesion were suspicious of a neoplasm. The main differential diagnoses at that point were pheochromocytoma, Cushing’s disease, primary hyperaldosteronism, and metastatic lesion. A blood panel comprising of 24-hour urine for free metanephrines and catecholamines, 24-hour urinary free cortisol, morning plasma renin activity, and aldosterone levels were requested. An early follow-up was requested with repeat CT scans and lab results. Her lab results came back negative for the aforementioned tests. She was advised to return to the Endocrine clinic in a year but unfortunately she was lost to follow-up.

A few years later, the patient presented to the hospital for abdominal pain and fever. She was treated for diverticulitis with antibiotic. Her abdominal CT scan showed the left adrenal lesion had increased to 9.6 cm x 7.7 cm in size.

The patient was referred to surgery where she underwent unilateral adrenalectomy. She was prophylactically given alpha-blocker treatment to prevent anesthesia and surgery related complications in pheochromocytoma. Patient did well perioperatively. Her surgical pathology showed an adrenal cortical carcinoma. It was low-grade, without lymphatic or vascular invasion. Tumor margins were negative and the tumor was confined to the adrenal cortex without invasion through the tumor capsule. No lymph nodes involvement was identified. There was no evidence of a pheochromocytoma on surgical pathology. The follow-up scans showed increased activity at the previous tumor bed and biopsy from interventional radiology showed remnant disease activity. At this point, patient sought care at the MD Anderson cancer center in Houston where she underwent repeat surgery for residual tumor resection and lymphadenectomy followed by mitotane treatment.

## DISCUSSION

Adrenocortical carcinoma (ACC) is an extremely rare tumor. In the U.S., the annual incidence of ACC is 0.72 cases per million population per year.^2^ A population-based study by Sharma et al. reported that ACC tumors have a female predominance, occur more commonly in whites and have the peak incidence in the sixth decade of life.^3^ Our case fits within these criteria as our patient was a Caucasian lady in her sixties.

Patients with ACC have a very poor prognosis with a 5-year overall survival (OS) below 30% in most cases. Surgical removal is often curative while others are slow to metastasize. Most ACCs occur sporadically, but some inherited disorders are associated more commonly with ACC, such as Li-Fraumeni syndrome (LFS), Beckwith-Wiedemann syndrome (BWS), Lynch syndrome (LS) and multiple endocrine neoplasia syndrome type 1 (MEN1).^4^

Prognosis is based on staging. In recent studies, patients with stage I disease had an estimated 5-year OS rate of 66% to 82% while patients with stage II and III disease had a 5-year OS rate that varied from 58% to 64% and 24% to 50%, respectively. In patients with stage IV disease, the 5-year OS rate decreased to 0% to 17%.

Reviews of the literature indicate that most adrenal incidentalomas are adenomas (80%), and only 8% are carcinomas. Compared to ACCs, pheochromocytomas or paraganglioma (PPGL) arises from the chromaffin cells of the adrenal medulla. Approximately 80 to 85% of chromaffin-cell tumors are pheochromocytomas, whereas 15 to 20% are paragangliomas.^5^ Combined, they are known as PPGLs.

The diagnosis of malignant PPGL relies on the presence of local invasion or metastasis and evidence of a metabolically active tumor as was seen in our case. The most common sites of metastasis include bones, lymph nodes and the liver.^6^

Overall, the prognosis is good as a recent retrospective study from the Mayo Clinic showed that the median overall and disease-specific survivals for malignant PPGLs were 25 and 34 years, respectively. These findings indicate a slow disease course in a subgroup of patients.^7^

Our case is interesting and extremely unique for a variety of reasons. First, pheochromocytoma is an extremely rare tumor in itself. The estimated incidence of this tumor in the general population is 2–8 cases per million/year.^8^ It is even rarer for an adrenal incidentaloma to present as a pheochromocytoma because only 5-6.5% cases of adrenal incidentaloma present clinically or biochemically as pheochromocytoma.

Second, the majority of the pheochromocytoma are symptomatic with a variety of symptoms. The most common symptoms of incidentaloma presenting as pheochromocytoma include palpitations, severe headache, persistent or paroxysmal hypertension, or abdominal pain. Only 8% of the pheochromocytoma present as totally asymptomatic tumors.^9^ The patient not only

had an ACC but showed biochemical features of pheochromocytoma as well as the urine was positive for metanephrine, epinephrine, and norepinephrine.

Finally, ACC tumors are aggressive tumors with distinct radiological and histological features. The radiological features suggestive of ACC include tumor calcification, irregular margins of the tumors, low fat-content, and presence of necrotic area. The histological features suggestive of ACC include areas of necrosis, venous invasion, capsular infiltration, and high-mitotic rate in the tumor.^10^ The adrenal mass did not show any radiological or histological features of traditional ACC.

## CONCLUSION

Adrenocortical adenocarcinoma (ACC) is a rare malignancy but it should be considered in all adrenal incidentalomas.
